# How does transmembrane electrochemical potential drive the rotation of F_o_ motor in an ATP synthase?

**DOI:** 10.1007/s13238-015-0217-6

**Published:** 2015-10-15

**Authors:** Xuejun C. Zhang, Min Liu, Yan Zhao

**Affiliations:** National Laboratory of Macromolecules, National Center of Protein Science-Beijing, Institute of Biophysics, Chinese Academy of Sciences, Beijing, 100101 China

**Table:** 

A REVISIT TO AN ANCIENT MOLECULAR MACHINE	
ATP synthesis via F_1_/F_o_-like ATP synthases is a universal energy conversion process in all living cells. An essential step of this process is to utilize the free energy of the transmembrane electrochemical potential of cations, such as protons or sodium ions, to drive the rotation of the F_o_ motor. Although membrane potential has been proposed to fuel the process for over ten years, this thermodynamic concept has not been firmly accepted in the ATP synthase research field partially because of lack of detailed mechanisms. In this article, based on theoretical considerations and in light of recent structural insights, Zhang and colleagues propose that the F_o_ rotation is driven by a noise-resistant thermodynamic process which converts the electrostatic free energy of the cations moving in the rotor-stator interface into the rotational kinetic energy. In this hypothesis, the shape difference between the interface-located trajectories in the rotator- and stator-reference systems ensures the unidirectional rotation of the F_o_ motor. This hypothesis offers new directions of combining experimental observations with thermodynamics and is a step further towards understanding the mechanism of the rotation of the F_o_ motor. —Jia-Huai Wang*	

The complexes of triphosphate adenosine (ATP) synthases belong to one of the most ancient protein families, and as such the complexes are ubiquitously distributed in all life kingdoms (Gruber et al., 2014; Nakamoto et al., 2008). Members of the ATP synthase family function as nano-turbines, possibly the smallest that exist in nature. Enzymes of this family catalyze either ATP synthesis or its reverse reaction, ATP hydrolysis. As implied by Peter Mitchell’s chemiosmotics theory developed in the 1960s, ion-translocating rotary ATPases serve either as ATP synthases, consuming energy supplied by transmembrane ion motive force (mostly from H^+^ or Na^+^) to generate ATP, or as transmembrane ion pumps powered by ATP hydrolysis. Because of the homology of the binding sites for proton and Na^+^ (Gruber et al., 2014), the same principles of energy transfer of proton motive force (PMF)-driven ATP synthases is likely to be applicable to Na^+^-driven ATP synthases as well. Moreover, some rotors of ATP synthases even switch between PMF and Na^+^ as their driving substance depending on the supply of either of these two types of ions. Therefore, for simplicity, here we will focus on PMF-driven ATP synthases. More importantly, each member of the ATP-synthase family contains two motor sectors: F_o_ couples proton translocation to rotation, and F_1_ couples rotation to ATP synthesis or hydrolysis (Fig. 1). While the F_1_ mechanism has been well established (Boyer, 1993), mechanisms of the F_o_ motor remain controversial. Although several models of converting the electrochemical potential energy of protons into the rotational kinetic energy of the F_o_ rotor have been proposed, a consensus remains to be established.

**Figure 1:**
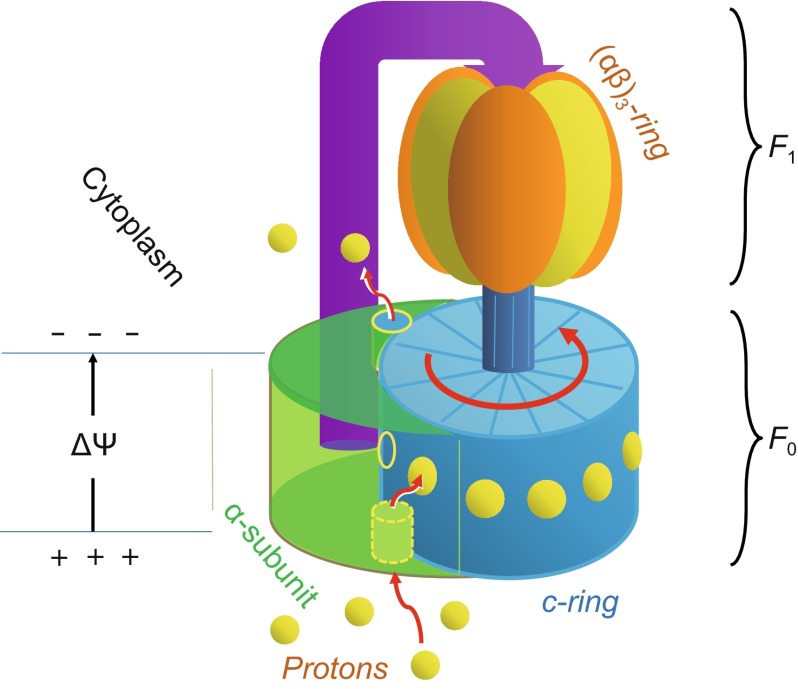
**Schematic diagram of the ATP synthase**

Recently, we proposed an electrostatic membrane potential (ΔΨ)-driving hypothesis in an attempt to unify energy-coupling mechanisms of a variety of transmembrane proteins into a common theoretical framework. The central idea of this hypothesis is that the interaction of charged groups in a membrane protein with ΔΨ can provide a major driving force for the functional cycle of the corresponding membrane protein. This hypothesis has been used to explain the energy coupling mechanisms of PMF-driven major facilitator superfamily (MFS) transporters (Zhang et al., 2015a), of proton transfer-mediated GPCR activation (Zhang et al., 2014), and of the ABC transporters (Zhang et al., 2015b). For example, in the MFS case, protonation of the transporter applies an extra electrostatic force, which together with the hydrophobic mismatch forces generates a torque to induce conformational changes for substrate transport. In this article, we propose the application of the same ΔΨ-driving hypothesis for identifying the mechanism of the functional cycle of the F_o_ motor. We will summarize the current knowledge on structures of ATP synthases and briefly discuss the current models of the mechanism of the F_o_ motor. One of the models, referred to here as the rigid-rotor model, will be further refined in light of newly available structural information and in the framework of our ΔΨ-driving hypothesis.

## AVAILABLE STRUCTURAL INFORMATION ON ATP SYNTHASES

Many ATP synthases and rotary ATPases from different species have been studied extensively both biochemically and structurally. For examples, a cryo-EM structure of the full-size PMF-driven ATP synthase from archaea *Thermus thermophiles* was reported at 9.7-Å resolution (Lau and Rubinstein, 2012), and most recently the cryo-EM structure of V-ATPase from yeast *Saccharomyces cerevisiae* was reported at 6.9-Å resolution (Zhao et al., 2015). While these protein complexes share the same overall three-dimensional organization of their major subunits, for historic reasons homologous subunits of these complexes are often named differently. In the following discussion, we will use nomenclatures for F_1_/F_o_ (i.e. fraction 1 and oligomycin-binding fraction) ATPases from *E. coli* and yeast mitochondria (Nakamoto et al., 2008).

It has been known from the early work of ATP synthase purification that each member of the ATP-synthase family contains a membrane associated F_o_ sector and a soluble F_1_ sector. The F_1_ sector may be located in places like cytosol, matrix of mitochondrial, or stroma of chloroplast, depending on the type of the ATP synthase. For the sake of discussion (without losing generality), we assume that the F_1_ sector is located in the cytosol (or simply the ‘inner’ space). This sector contains subunits (αβ)_3_γ_1_δ_1_ε_1_, and its major structural feature is a pseudo three-fold symmetrical (αβ)_3_ complex (i.e. the yellow-orange ring in Fig. 1) (Abrahams et al., 1994). The membrane-embedded F_o_ sector contains subunits *a*_1_*b*_2_*c*_nc_ (where n_c_= 8–15), and its core consists of a symmetrical (typically decameric) *c*-ring formed by *c*-subunits (Stock et al., 1999; Fillingame and Steed, 2014). The two motor sectors are coupled mechanically through a common central shaft. Due to the coupling, some subunits from both F_o_ and F_1_ sectors form a stable complex (i.e. α_3_β_3_δ_1_ +a_1_b_2_), the so-called stator, while the remaining subunits (i.e. γ_1_ε_1_ +c_10_) form the rotor. Although functions of the ATP synthase only require the relative rotation between the stator and rotor, it is commonly assumed that the stator is stationary relatively to the membrane, while the rotor rotates inside the membrane. In eukaryotic V-type ATPases, the stator is found to link structurally with the intracellular actin network (Holliday et al., 2000), thus indeed being immobilized to the membrane.

The rotation dynamics of the F_1_ sector has been demonstrated with single-molecule studies (Noji et al., 1997; Minagawa et al., 2013). For example, the results from V_1_-ATPase of *E. hirae* indicate that, during sustained ATP hydrolysis, the central shaft rotates unidirectionally with a rotation rate over 100 revolutions per second (Minagawa et al., 2013). The theory of ATP synthesis in the F_1_ sector, through a mechanism of an asymmetric three-step rotation, has been well established (Nakamoto et al., 2008). Rotation of the central shaft from the rotor is accompanied by conformational changes in the β subunits of the F_1_ stator. These conformational changes cycle sequentially through functional states of low, medium, and high nucleotide affinities, corresponding to release of product (ATP), binding of substrates (ADP and P_i_), and ATP formation, respectively. This cycle is known as the “binding change mechanism” of ATP synthesis (Boyer, 1993). When being driven by ATP hydrolysis, the direction of the rotation of the central shaft relative to the immobilized F_1_ sector was observed to be counter-clockwise, viewed from the F_o_-proximal side (Noji et al., 1997). Thus, when being powered by PMF, the direction of the rotation of the *c*-ring in F_o_ will be in the opposite direction. In other words, if viewed from the F_1_-proximal/cytosol side, the rotation of the *c*-ring is also counter-clockwise (Fig. 1). Since the study of mechanism of the F_1_ motor is a well-established field, in the following we will focus on the mechanism of the F_o_ motor.

## CURRENT MODELS OF ENERGY CONVERSION IN F_O_ ROTORS

Currently, two major models co-exist for how F_o_ motors are driven by PMF. The first hypothesis, i.e. the rigid-rotor model, was originally proposed by Junge et al. (Junge et al., 1997). In this model, both the F_o_ rotor (i.e. the *c*-ring) and the corresponding stator (the *a*-subunit) are assumed to be rigid bodies individually. The mechanistic model argues that the relative motion between the rotor and stator is Brownian in nature, and this thermal rotational fluctuation is biased by PMF to go in the ‘correct’ direction, via a putative ratchet apparatus (Junge et al., 1997). In other words, thermodynamic rotatory fluctuation was assumed to be the dominant factor for energy conversion, while ΔΨ played only an assisting role in Junge’s original proposal. Subsequently, this problem was partially fixed by Dimroth et al. (2003). In particular, ΔΨ was explicitly considered as a major source of the thermodynamic driving energy of the F_o_ rotation. Nevertheless, the precise mechanisms for energy coupling remain to be elucidated. The second model, referred to here as a flexible-rotor model, was proposed more recently by Fillingame and co-workers, on the basis of their cryo-EM structure of *T. thermophiles* ATP synthase, together with data from cross-linking experiments (Fillingame and Steed, 2014). The main difference of this model to the first model is that the interface between the *a*-subunit and *c*-ring (i.e. rotor-stator interface) undergoes a precisely programmed multi-step rearrangement during the functional cycle of F_o_. An extensive, dynamic, inter-subunit network of side chain-side chain interactions is assumed to form the basis of concerted rotations of multiple transmembrane (TM) helices on both sides of the interface. Both the rigid- and flexible-rotor models hypothesize that the protons enter the F_o_ complex via a channel buried in the rotor-stator interface from the ‘outer’ space halfway into the membrane, and later leave the complex from a putative channel located inside the cytosolic/inner half of the stator (*a*-subunit) (Fig. 1). In particular, the second model is also explicitly called “two half-channel” model (Fillingame and Steed, 2014). Thus, the energy coupling mechanism of the F_o_ motor remains to be debated.

While essentially agreeing with the rigid-rotor model, we will argue that a deterministic motion, rather than Brownian motion, is the basis of a robust and highly efficient F_o_ rotor. In addition, the primary sequences of both *a-* and *c-*subunits of F_o_ are not conserved in general (Gruber et al., 2014). Yet, their functions, including the unidirectional rotation of the *c*-ring, have remained unchanged throughout evolution. Therefore, the energy-coupling mechanism of F_o_ rotation is more likely driven by a noise-resistant thermodynamic process, rather than by a precisely defined dynamic interaction network as proposed by the flexible-rotor model. For an ancient protein complex like ATP synthase that is essential to all cells, a simple and noise-resistant mechanism might be of particular importance in the early stages of evolution. In the following, we hope to present such a conceptually simple and practically robust mechanism for the F_o_ motor.

## THEORETICAL ARGUMENTS ON HOW PMF IS LINKED TO ROTOR POWER

To power the F_o_ motor of an ATP synthase, electrochemical potential energy of the driving substance, e.g. protons, needs to be converted into the rotational kinetic energy of the rotor. In principle, movements of electric charges along the direction of the electrostatic field of ΔΨ have the capacity to generate mechanical energy for the host proteins. The charged groups, e.g. H^+^ or Na^+^, function as the driving substance, i.e. the energy-carrying medium, analogous to the water in a hydroelectric dam and the steam in an engine. This view has been convincingly presented before in thermodynamic terms by Dimroth and others, who explored the energy source of the F_o_ motor (Dimroth et al., 2003; Nakano et al., 2006). The ΔΨ-associated free energy (FΔΨ, where F is the Faraday constant), which can be independent of ΔpH (i.e. pH difference between the extracellular space and the cytosol), is usually stronger than the free energy term associated with ΔpH (i.e. Δμ([H^+^]) = −2.3RTΔpH, where R is the universal gas constant and T is the absolute temperature). Intriguingly, there appears a reciprocal correlation between the number of *c-*subunits in the *c*-ring (thus the consumption of protons) and strength of ΔΨ (but not ΔpH) (Dimroth et al., 2003). In other words, in the presence of a stronger electrostatic membrane potential ΔΨ, a F_o_ motor often requires less protons (i.e. smaller n_c_), thus consuming roughly the same amount of energy (n_c_FΔΨ) as those F_o_ motors working under a weaker ΔΨ, to generate three ATP molecules in a complete functional cycle of the ATPase. This observation suggests that ΔΨ has a more profound effect on the function of the F_o_ motor than ΔpH. Nevertheless, ΔpH contributes to rotation of the *c*-ring as well. First, Δμ([H^+^]) is involved in the loading and releasing of protons, with energy terms ΔG_L_ and ΔG_R_, respectively (see Supplementary Material). The more negative these two terms are, the higher the probability that the loading and releasing steps occur spontaneously. However, both ΔG_L_ and ΔG_R_ terms eventually contribute to the heat release of the process, rather than to the mechanical energy of the *c*-ring rotor. Therefore, in order to maintain a high efficiency of energy conversion in the ATP synthase, the amount of released heat, thus the energy terms ΔG_L_ and ΔG_R_, must be kept small. Second, the remaining energy of Δμ([H^+^]), termed as differential binding energy ΔG_D_ between the loading and releasing states, may partially fuel the rotation (if ΔG_D_ < 0). One possible way of utilizing ΔG_D_ is for the protein complex to form a proton path consisting of sites of increasingly stronger affinities, thus promoting proton movement. However, with either ΔΨ or ΔpH, the mechanisms of converting linear movement of the proton in a given *c-*subunit to a rotation of the entire *c*-ring remain to be uncovered.

Membrane potential (ΔΨ) directly applies an electrostatic force on any electric charge (e.g. a proton bound to a titratable residue) in a membrane protein. Such interactions between ΔΨ and bound protons have been proposed to drive inter-domain rotations in PMF-driven MFS transporters with a so-called rocker-switch mechanism (Zhang et al., 2015a). It is noteworthy that the rotation axis in an MFS transporter is parallel to the membrane plane, rather than perpendicular to the plane as found in the F_o_ motor. What we attempt to do here is to identify the mechanisms of powering rotation of the F_o_ rotor with a force applied along the axial direction. The energy conversion in the F_o_ motor is conceptually similar to the conversion of gravitational potential energy into a rotational movement when an object is sliding down a spiral slide (e.g. in a children’s playground). Of course, another analogy is the wind turbine, which converts directional/linear air pressure into the rotation of the vane wheel. Such a rotational movement can be described in either the stationary reference system (termed as the S-system) or the rotor reference system (i.e. the R-system). In the spiral slide case, the trajectory in the S-system is a spiral curve, while the trajectory in the R-system is a straight line along the gravity. In contrast, in the wind turbine case, the trajectory of the air flow in the S-system appears to be a straight line along the wind direction, while that in the R-system appears to be a spiral curve. Thus, the difference between trajectories in the two reference systems is a fundamental feature of any rotation system. Understanding how the stator and rotor generate distinct trajectories for the proton movement appears to be the key to delineating the mechanism of maintaining a unidirectional rotation of the F_o_ rotor.

In contrast to the earlier rigid-rotor model, we argue that the driving substance, e.g. protons, must stay in the rotor-stator interface where its free energy is converted into the kinetic energy of the rotor. Roughly speaking, the rotational direction of a rigid rotor is determined by the torque that the rotor is subjected to. Mathematically, the torque (*T*) can be written as the differentiation of the Gibbs free energy (*G*) of an individual *c-*subunit relative to the rotation angle (θ):

1 $$T = -\frac{{d{\text{G}}(\uptheta)}}{d\uptheta}$$

The negative sign in equation 1 indicates that generation of *T* is associated with decrease in G(θ). For a complete rotation cycle of the c-ring, the change in G(θ) is n_c_FΔΨ, which is in the order of 4n_c_RT assuming a 100-mV ΔΨ. The key question here is how to make the Gibbs free energy monotonically descending in respect to the rotation angle, θ. It is clear that not all components of the Gibbs free energy are θ-dependent. For example, the above-mentioned free energy terms ΔG_L_ and ΔG_R_ are unlikely to contribute to *T*. As ΔΨ is the dominant component of the θ-dependent Gibbs free energy, changes of G(θ) occur only when the proton moves along the ΔΨ direction. In particular, each *c-*subunit is subjected to a torque (~ n_c_FΔΨ/2π) upon it moves into the vicinity of the *a-*subunit where the proton binding to the *c*-subunit is disturbed by the *a*-subunit. At other angular positions, G(θ) of the given *c-*subunit remains constant (thus resulting in a zero *T*) because of the uniform lipid environment as well as tight binding of the proton. In this “zero-*T*” time interval, the driving energy to the *c*-ring is provided by other *c-*subunits, which are moving into and leaving from the rotor-stator interface. Taken together, a unidirectional rotation would occur naturally if the rotation of the F_o_ rotor is accompanied by a decrease of G(θ).

**Figure 2:**
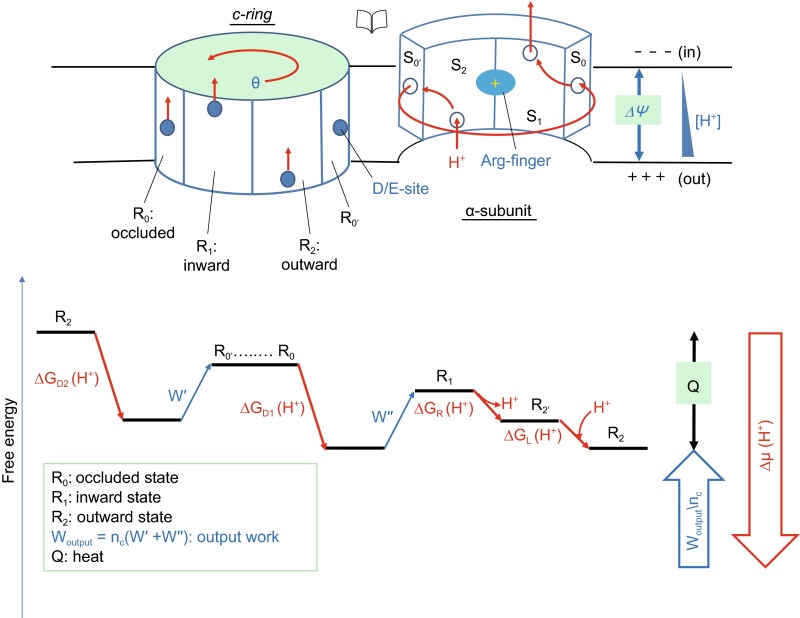
**Putative mechanism of PMF-driven movement of the** ***c*** **-ring**. Schematic diagram of the rotor-stator interface shown in an open-book view. Putative high p*K*_*a*_ sites in different states in the *c-*ring are marked with solid blue circles. The corresponding sites in the *a-*subunit are marked with open blue circles. Direction of the electrostatic force applied to the bound protons is marked with solid red arrows. The proton path in the stator reference system (S-path) is marked with red dash lines. The intracellular space where the ATP synthesis occurs is labeled as ‘in’, and the opposite side of the membrane as ‘out’

As we discussed above, the shape of the proton trajectory plays important roles in energy conversion. For the sake of discussion, we assume that for a given *c-*subunit the proton trajectory in the R-system (termed as R-path) is a straight line from the extracellular side to the cytosolic side of the membrane. Meanwhile, the proton path in the S-system (termed as S-path) takes an overall right-hand spiral shape, as shown in Fig. 2. Thus, the F_o_ motor works in a manner more similar to the above-mentioned spiral slide. As the two reference systems rotate relative to each other, the cross point of the two proton paths becomes the current proton-binding site in the rotor-stator interface. Each of these proton binding-sites along the trajectory is formed by both a- and c- subunits. Alternatively, these sites may be alternately distributed on both subunits. In other words, viewed in the S-system, the structure of the interface prohibits a movement of the proton in a straight line parallel to R-path. The ΔΨ-driven movement of the proton would be halted unless the *c*-ring rotates into the next position. In spite of the above assumption of a straight path, the exact shape of the R-path is not critical for the unidirectional F_o_ rotation, provided that the next proton-binding site along the R-path lags behind that of the S-path. This positional difference between the two paths is the basis of the θ-dependency of G(θ), so that the *c*-ring rotation will make the R-path catch up with the S-path as the proton moves along the ΔΨ direction. Furthermore, in the S-system the direction of the proton movement (thumb) and the rotation of the spiral path (fingers) follow the right-hand rule. The lag of the R-path behind the S-path, in the context of the overall right-hand spiral shape of the S-path, is essential for a monotonically descending G(θ) function, and thus for the smooth, unidirectional rotation of the *c*-ring.

It is important to emphasize that the proton path must be physically located in the rotor-stator interface during the energy converting, because the torque is to be applied between the rotor and the stator. Once the proton moves away from the *c-*ring, its movement becomes irrelevant to the *c-*ring rotation. However, in nearly all the previous models, the proton is assumed to enter a putative half-channel in the stator before it is released into the cytosol. Such a half-channel might work as an exit for the proton only if the channel was large enough to carry sufficient solvent, which has a much higher dielectric constant than the lipid bilayer or the protein. In this scenario, the electric field of the ΔΨ in this half-channel would diminish essentially to zero, thus proton movement within this channel would not be powered by ΔΨ but as free diffusion. In short, no significant amount of free energy of the proton should be wasted in the putative half-channel inside the stator.

## STRUCTURAL EVIDENCE TO SUPPORT THE REFINED RIGID-ROTOR MODEL

Recent progresses in structural studies on ATP synthases provide important information for solving the mystery of how F_o_ rotation is coupled with the decrease of the free energy of the protons. Cryo-EM structures of both *T. thermophiles* ATP synthase and *S. cerevisiae* V_1_/V_o_-ATPase revealed the presence of interactions between the *c*-ring and *a-*subunit (Lau and Rubinstein, 2012; Zhao et al., 2015). The *a-*subunit contains two long and highly titled TM helices, which contact two adjacent *c*-subunits, yet with distinct interactions. In addition, all subunits in the *c*-ring show a uniform conformation (at the resolution supported by EM) regardless of their relative positions to the *a-*subunit. However, based on their distinct micro-environments, the multiple *c-*subunits can be classified into three states, namely R_0_, R_1_, and R_2_ (where R stands for rotor; Fig. 2). While R_0_ is the state in which the *c-*subunit faces the lipid bilayer (thus away from the *a-*subunit), R_1_ and R_2_ are the states in which the *c*-subunit contacts the *a-*subunit. For ATP synthesis, a given *c-*subunit in the *c*-ring cycles through different states in the sequential order of R_0_, R_1_, R_2_, and R_0_ again. Alternatively, the rotor-stator interface can be divided into S_0_, S_1_, S_2_, and S_0’_ regions (where S stands for stator; Fig. 2), corresponding to the *c-*subunits in their respective states. From this latter view, connecting proton-binding sites from sequential *c-*subunits result in an overall right-hand spiral-like S-path, as we proposed above based on theoretical consideration. As each *c-*subunit contains one and only one proton-binding site at any given moment, the energy associated with creating a new binding site is compensated by the energy of removing an old site. In other words, energy difference between the states, R_0_, R_1_, and R_2_, is solely determined by the proton position relative to membrane (or ΔΨ). Together, these recent cryo-EM structures allowed for the first time to identify two distinct binding modes between the *a*-subunit and two neighboring *c*-subunits, thus providing structural evidence of distinct loading and releasing states for the protons.

## ROLES OF D/E-SITE AND Arg-FINGER IN CHANGING THE STATUS OF PROTON-BINDING SITES OF THE *c*-RING

Similar to previous models, our above-proposed mechanism of F_o_ rotation requires a stable proton-binding site in the R_0_ state of the *c*-subunit. The proton-affinity of this binding site must increase upon moving out of the rotor-stator interface and decrease upon moving into the interface. Such a proton-binding site is evident from many of the previously reported crystal structures of *c*-rings (Stock et al., 1999; Symersky et al., 2012; Pogoryelov et al., 2009). In the middle of the TM region of each *c-*subunit, there is a conserved acidic residue (referred to here as D/E-site), e.g. cAsp61 in *E. coli* and cGlu59 in *S. cerevisiae*, that is protonated and buried in the interface between neighboring *c-*subunits in the isolated *c*-ring. This D/E-site is the protonation site in the R_0_ state. Furthermore, there is a conserved Arg residue in the *a-*subunit, e.g. aArg210 in *E. coli* F_o_ (Lightowlers et al., 1987) (referred to here as Arg-finger). Although its position has not been precisely determined in the cryo-EM structures, this Arg-finger is believed to be located in the middle of TM4 of the *a-*subunit, facing the D/E-site of the *c*-ring in either the S_1_ or S_2_ states or both (Lau and Rubinstein, 2012; Fillingame and Steed, 2014). Both the D/E-site and Arg-finger have been shown to be essential for the activity of F_o_ (Miller et al., 1990; Valiyaveetil and Fillingame, 1997). An interaction of the D/E-site with the Arg-finger necessarily decreases the p*K*_*a*_, thus promoting deprotonation from the D/E-site. Moreover, in the S_1_ region of the rotor-stator interface, there is a putative half-channel for proton translocation from the middle part of the transmembrane region to the cytosol/inner space. Thus, R_1_ is considered as an inward-connecting state. In contrast, in the R_2_ state the D/E-site is assumed to connect with the ‘outer’ space through another half-channel, and thus R_2_ is considered as an outward-connecting state. In addition, the R_0_ state is where the D/E-site becomes protonated and buried in a hydrophobic environment, and thus R_0_ can be considered as an occluded state. During the rotation of the *c*-ring, the microenvironment of the D/E-site of a given *c-*subunit cycles between these states, by changing interaction with the *a-*subunit. In particular, right before moving into the hydrophobic lipid bilayer, the D/E-site obtains a proton from the ‘outer’ space, being pushed by ΔΨ. After a nearly complete cycle of rotation, the D/E-site approaches the Arg-finger and becomes deprotonated. The released proton, in turn, is pushed into the ‘inner’ space, again by ΔΨ. Therefore, the Arg-finger seems to play two roles: First, it serves as a trigger of the proton release from the D/E-site to the cytosol when the *c*-subunit moves into in the S_0_-S_1_ region; and second, it serves as a ratchet in the S_2_-S_0’_ region to prevent backward rotation. Should a backward rotation occur, the protonated D/E-site would have to eject its bound proton backwards to the ‘outer’ space due to the existence of the positive charge of Arg-finger. Such an event would be against the membrane potential and thus be energetically costly. Therefore, a backwards movement only occurs in the process of ATP hydrolysis-driven proton pumping, not however, during ATP synthesis. In support of the refined rigid-rotor model following our ΔΨ-driving hypothesis, an *E. coli* aR210A variant showed proton leakage and abolished coupling between ATP hydrolysis in F_1_ and proton translocation in F_o_ (Valiyaveetil and Fillingame, 1997). These defects are most likely related to a backward rotation of the *c*-ring instigated by the aR210A mutation. In addition, a non-functional aR210Q mutation can be rescued by an aQ252R substitution in TM5 of the *a-*subunit (Valiyaveetil and Fillingame, 1997). This observation suggests that aGln252 is spatially close to the Arg-finger, and the introduced aArg252 residue takes the role of aArg210 in maintaining the unidirectional *c*-ring rotation. Since electrostatic interaction functions in long range, the deprotonation of the D/E-site does not require a direct contact with the Arg-finger via a salt-bridge bond. Thus, the previously proposed concerted rotations of TM helices in both the *a-* and *c-*subunits (Fillingame and Steed, 2014) and the exposing of D/E-site (cAsp61) (Symersky et al., 2012) become unnecessary according to our refined model, although the packing of TM helices of the *a-*subunit from some species may indeed be flexible. In agreement with this argument, displacing the D/E-site from one TM helix to another in the *c*-subunit has been found to result in a partially functional *c*-ring (Miller et al., 1990). Moreover, formation of a salt-bridge bond per se between the D/E-site and Arg-finger would neither energize the rotation of the *c*-ring nor determine its directionality, since the same energy released during the bond formation must be paid back when the bond is broken. Taken together, the Arg-finger in the *a-*subunit shapes the trajectory of the protons in the F_o_ rotor by dynamically regulating the proton affinity of the D/E-site in the *c-*subunit.

## WHAT PROPERTIES SHOULD THE DYNAMIC PROTON PATH HAVE?

As one of the core concepts of our refined mechanistic model of the F_o_ motor, a dynamic proton-wire in the rotor-stator interface is essential for the function of F_o_. Here, we discuss how this proton-wire is formed, based mainly on the insights obtained from structural analyses. The high efficiency of energy-conversion of the F_o_ motor (and thus that of the ATP synthase (Silverstein, 2014)) requires most of the free energy of the transported protons be converted to the rotary kinetic energy of the rotor in a step-wise manner. Otherwise, if the protons were shut out from the F_o_ rotor into the ‘inner’ space (or into the stator) carrying significant amount of kinetic energy, the loss of a large amount of energy in the form of heat would be inevitable (see Fig. S1). Thus, high efficiency of energy conversion requires the existence of a series of proton-binding sites of controllable affinities along the proton path, to absorb sequentially the kinetic energy of the proton from each step of the movement. Interestingly, on the surface of the c_10_-ring of yeast mitochondrial F_o_ (PDB ID: 3U2F, 2.0 Å) (Symersky et al., 2012) and that of the c_15_-ring of *Spirulina platensis* chloroplast F_o_ (PDB ID: 2WIE; 2.1 Å) (Pogoryelov et al., 2009), there is a hydrophilic surface groove formed by the peptide planes of the C-terminal TM helix of each subunit c. This groove faces the lipid bilayer and passes by the D/E-site. In the crystal structure of *S. platensis* c_15_-ring structure, the thylakoid-space half of this groove is covered by a string of small pieces of electron densities, which were modeled as the terminal C_6_-ring of Cymal-4 detergent, mimicking a water channel. It is known that, in many coiled-coil helix structures from soluble proteins, the backbone hydrogen bonds of α-helices are often accompanied by water molecules bound in surface grooves (see examples in PDB file 2Q12 (Zhu et al., 2007) and 3CI9 (Liu et al., 2009)). Thus, the surface grooves observed in the *c*-ring structures may provide binding sites for water molecules (or H_3_O^+^), and these putative bound water molecules may form part of a proton-wire, running from the extracellular side to the cytosol side. An investigation into the relationship between such putative hydronium binding sites on the surface of the *c-*ring with previously proposed H_3_O^+^-mediated proton-translocation (von Ballmoos and Dimroth, 2007; Boyer, 1988) deserves further attention. In particular, in his 1988 review (Boyer, 1988), Boyer pointed out that “[c]arbonyl oxygens of peptide bonds can coordinate a single chain of water molecules, forming a continuous phase along which protons can travel, analogous to the rapid migration of protons in ice”. The structural information obtained after this landmark review provides support to this hypothesis. For such a putative, water-mediated proton-wire to function, it is not necessarily the water molecules per se that move along with the protons. Rather, the water molecules may either stay in the groove or transiently bind to it when the groove faces the *a-*subunit. The affinity of such proton-binding sites may be further dynamically regulated by the interactions in the rotor-stator interface. For instance, it is possible that this proton-wire in the *c-*subunit is discontinuous here and there, and thus residues from the *a*-subunit are needed to complete the proton path, in a manner analogous to gear wheels, during the rotation of the *c-*ring. By doing so, a dynamic right-hand spiral curve of the S-path can be realized in the rotor-stator interface.

## SUMMARY

While the field of ATP synthase research has a long history filled with landmark discoveries, recent structural works provide us with important insights into the mechanisms that links the proton movement with the rotation of the F_o_ motor. Here, we propose a mechanism of unidirectional rotation of the F_o_ complex, which is in agreement with these new structural insights as well as our more general ΔΨ-driving hypothesis of membrane proteins: A proton path in the rotor-stator interface is formed dynamically in concert with the rotation of the F_o_ rotor. The trajectory of the proton viewed in the reference system of the rotor (R-path) must lag behind that of the stator (S-path). The proton moves from a higher energy site to a lower site following both trajectories simultaneously. The two trajectories meet each other at the transient proton-binding site, resulting in a relative rotation between the rotor and stator. The kinetic energy of protons gained from ΔΨ is transferred to the *c*-ring as the protons are captured sequentially by the binding sites along the proton path, thus driving the unidirectional rotation of the *c*-ring. Our ΔΨ-driving hypothesis on F_o_ motor is an attempt to unveil the robust mechanism of energy conversion in the highly conserved, ubiquitously expressed rotary ATP synthases.

## Electronic supplementary material

Supplementary material 1 (PDF 193 kb)
