# Supplementary material for: How does transmembrane electrochemical potential drive the rotation of Fo motor in an ATP synthase?
Source: Protein Cell. 2015 Oct 15;6(11):784–91. doi: 10.1007/s13238-015-0217-6 (PMC4624678; doi:10.1007/s13238-015-0217-6)
Supplement: Supplementary file 1 — Supplementary material 1 (PDF 193 kb) [file 13238_2015_217_MOESM1_ESM.pdf]

Supplementary Material

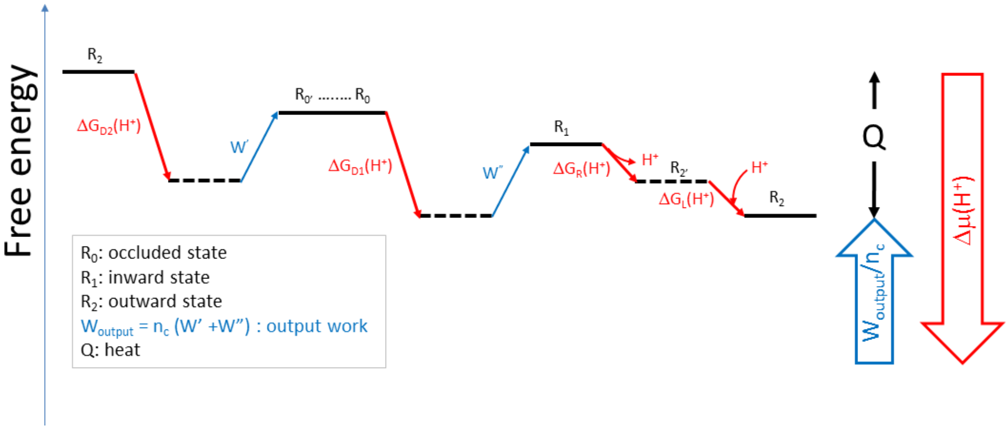

Figure A1. Schematic plot of energy landscape of the F_o motor.

Free-energy landscape plot describing the thermodynamic relationship between different states. Horizontal lines represent states, with imaginary intermediate states in dashed lines. Tilted lines represent transitions between states. Locally, any transition of positive ΔG must be driven by a neighboring transition of a negative ΔG. Components in blue are related to output energy of the motor, and those in red to PMF. Because the transport process is cyclical, the choice of the starting point is arbitrary. Therefore, the starting and ending states are identical, only differing in the release of heat (Q) during one transport cycle. On one hand, this heat release can be considered as a thermodynamic driving force for the process: The larger it is, the faster the process may occur. On the other hand, in order to achieve energy conversion of high efficiency, Q should be small, approaching zero.

Free energy terms associated with proton translocation in F_o complex

See Figs. 2 and A1 for illustrations. A negative ΔG indicates that the corresponding process is thermodynamically favorable.

$$n_c\Delta\mu(H^+) + W_{output} = -Q < 0$$

(Second law of thermodynamics. Efficiency of energy conversion is $W_{output} / |n_c\Delta\mu(H^+)| < 1$)

$$\Delta\mu(H^+) \equiv F\Delta\Psi + \Delta\mu([H^+]) < 0$$

(electrochemical potential of proton; where $\Delta\Psi < 0$)

$$\Delta\mu([H^+]) \equiv RT\,ln([H^+]_R/[H^+]_L) = -2.3RT\Delta pH$$

$$= \Delta G_L(H^+) + \Delta G_R(H^+) + \Delta G_D(H^+) < 0$$

(chemical potential of proton concentration; where $\Delta pH > 0$)

$\Delta G_L(H^+) \equiv RT\,ln(K_{d,2}/[H^+]_L)$

(free energy of proton loading from the extracellular/‘outer’ space)

$\Delta G_R(H^+) \equiv RT\,ln(\,[H^+]_R/K_{d,1})$

(free energy of proton releasing to the cytosolic/’inner’ space)

$\Delta G_D(H^+) \equiv RT\,ln(K_{d,1}/K_{d,2})$

(differential binding energy of proton between S_1 and S_2 states)

$\Delta G_{D1}(H^+) + \Delta G_{D2}(H^+) = F\Delta\Psi + \Delta G_D(H^+)$

(driving energy for the rotation)

$\Delta G_{D1}(H^+) \equiv \gamma F\Delta\Psi + RT\,ln(K_{d,1}/K_{d,0})$

(driving energy between R_0 and R_1 states)

$\Delta G_{D2}(H^+) \equiv (1-\gamma)F\Delta\Psi + RT\,ln(K_{d,0}/K_{d,2})$

(driving energy between R_2 and R_0 states)
